# The influence of climate on peatland extent in Western Siberia since the Last Glacial Maximum

**DOI:** 10.1038/srep24784

**Published:** 2016-04-20

**Authors:** G. A. Alexandrov, V. A. Brovkin, T. Kleinen

**Affiliations:** 1 A. M. Obukhov Institute of Atmospheric Physics, Russian Academy of Sciences, Pyzhevsky 3, Moscow, 119017, Russia; 2 Max Planck Institute for Meteorology, Bundesstrasse 53, 20146 Hamburg, Germany

## Abstract

Boreal and subarctic peatlands are an important dynamical component of the earth system. They are sensitive to climate change, and could either continue to serve as a carbon sink or become a carbon source. Climatic thresholds for switching peatlands from sink to source are not well defined, and therefore, incorporating peatlands into Earth system models is a challenging task. Here we introduce a climatic index, warm precipitation excess, to delineate the potential geographic distribution of boreal peatlands for a given climate and landscape morphology. This allows us to explain the present-day distribution of peatlands in Western Siberia, their absence during the Last Glacial Maximum, their expansion during the mid-Holocene, and to form a working hypothesis about the trend to peatland degradation in the southern taiga belt of Western Siberia under an RCP 8.5 scenario for the projected climate in year 2100.

Boreal and subarctic peatlands are widely considered an important dynamical component of the earth system. The reason is twofold. First, they store a large amount of carbon −500 ± 100 GtC^1^, 20*–*40% of total soil organic carbon of the world^2^. Second, they are sensitive to climate change^3^, in that even minor changes in climate regime may turn them into a major CO_2_ source^4^. The vulnerability of carbon stocks accumulated by peatlands, and the related climate feedbacks, have been intensively researched for a number of years^5^,^6^,^7^,^8^,^9^,^10^,^11^, but incorporating peatlands into Earth system models^12^,^13^,^14^,^15^ still remains somewhat challenging: climatic thresholds for switching between peatland expansion and peatland degradation are not well defined.

The absence of peatlands in Western Siberia during the Last Glacial Maximum (LGM)^16^ is one of the puzzles waiting to be solved. At present, the territory of Western Siberia is densely covered by peatlands^17^. However, very few of them initiated between 18,000 and 11,000 years before present (BP): mostly, paludification of Western Siberia started 11,000 years BP^18^. This late start of peatlands expansion was not seen as a puzzle several decades ago, because it was wrongly assumed then that an ice sheet covered Western Siberia. However, more recent findings show that Western Siberia was in fact ice-free during the last glacial^19^. Simulations of LGM climate performed with the Max Planck Institute - Earth System Model (MPI-ESM)^20^,^21^ suggest, as one can see in Fig. 1, that climatic conditions were neither extremely dry nor extremely cold. In terms of Holdridge life zones^22^, they were similar to moist tundra or moist boreal forest for the major part of Western Siberia. Peatlands are common in these life zones at present^23^.

Then, what did prevent peatland expansion during the LGM?

We suppose that summer dryness was the key factor. *Sphagnum*-dominated peatlands are sensitive to the moisture balance: in these ecosystems, the waterlogged peat accumulates above the level of the surrounding drainage network, and waterlogging is maintained through impeded drainage^24^. Therefore, an excess of summertime precipitation is certainly needed to keep the moisture balance.

The autogenic hydrological feedbacks that make northern peatlands resistant to seasonal water deficit^25^,^26^ are efficient within a certain range of climatic conditions. This range can be assessed based on the graphical summary of observations provided by Verry^27^ that relates actual evapotranspiration (AET) to water table depth. Since AET declines with lowering water table, a decrease in precipitation has less effect on the water table depth than it could have otherwise. However, AET does not decline to zero but remains at 70% of potential evapotranspiration (PET), when water table depth drops to the level at which stable *Sphagnum* growth becomes doubtful (ref. 27). Therefore, we assume that 0.7PET is the precipitation threshold for peatland expansion. This enables us to introduce a new index for “measuring” climate suitability for peatland expansion: warm precip excess (WPE), which is defined as *P*_w_ − 0.7PET, where *P*_w_ is precipitation during the warm period of the year.

The fraction of land that could be occupied by peatlands depends not only on climatic conditions. The lateral expansion of peatlands is driven by local hydrological processes closely linked to landscape morphology^28^. Therefore, peatlands may not become geographically widespread until WPE exceeds a certain threshold set by landscape morphology.

To find this WPE threshold we apply a model based on the Dupuit-Forchheimer theory of groundwater movement^29^ (see *Methods*). This theory, starting from the seminal work of Ingram (ref. 24), is often used to simulate raised mire growth^30^,^31^,^32^. It allows us to estimate the fraction of watershed area where waterlogging is maintained through impeded drainage. As climate input we use the fields of mean monthly temperature and precipitation simulated by MPI-ESM (refs 20, 21) for present-day, mid-Holocene, LGM, and 2100 climate.

## Results

**Figure 2:**
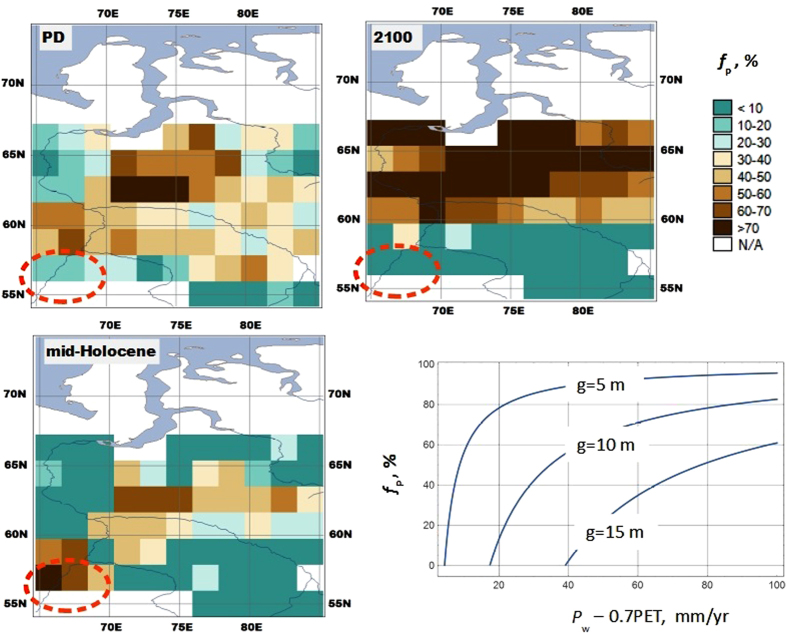
The fraction of land that could be occupied by peatlands under present climate (upper left corner), mid-Holocene climate (lower left corner), and climate in 2100 following the RCP 8.5 scenario (upper right corner), and for a given value of WPE (lower right corner), when P/PET = 1.2, and the length of the warm period is 5 months. The dashed orange line encircles the area within the Tobol river basin where *f*_P_ simulated for mid-Holocene climate exceeds the present level. The fraction of land that could be occupied by peatlands under LGM climate is not shown, because it has spatially uniform value that equals zero. Made with *MapWindow* 4.8.8 (http://www.mapwindow.org/), *Natural Earth* public domain map data (http://www.naturalearthdata.com/about/terms-of-use/), and map colors from www.ColorBrewer.org.

The impeded drainage model (IDM) implies that the critical threshold of WPE, beyond which the expansion of peatlands is started, increases with the averaged watershed elevation above the level of the drainage system, *g*. The higher is *g*, the higher is the threshold. We estimated its values for grid cells of MPI-ESM from the present geographic distribution of peatlands (ref. 17). Optimized *g* values, that is, the values that make IDM fit data on the fraction of land covered by peatlands, vary between 2.6 and 19.6 m, that is, around 10 m. At *g* = 10 m, half of the watershed area is suitable for peatland expansion when WPE is equal to 30 mm/yr (Fig. 2, lower right corner). Therefore, 30 mm/yr could be considered a level of WPE that “turns on” the process of massive peatland expansion in Western Siberia. This is, of course, a very coarse estimate. It does not take into account the spatial diversity of climatic conditions, and therefore, its use is limited to continental scale, e.g., for delineating climatic regions where peatlands may theoretically occur.

Under LGM climate conditions, WPE is negative almost everywhere in Western Siberia. In those grid cells where WPE is positive (see Supplementary Information), it is still below the threshold set by landscape morphology. Therefore, the optimized IDM gives a spatially uniform value of *f*_P_ = 0 for LGM climate. According to the model, peatlands were not widespread in Western Siberia during the LGM because the summers were too dry for their initiation.

Applying the optimized IDM to the present day climate we naturally came to the fraction of land suitable for peatland expansion (*f*_P_) that mimics the contemporary geographic distribution of peatlands (Fig. 2, upper left corner). In the case of the mid-Holocene climate, the optimized IDM gives a slightly different geographic pattern of *f*_P_ (Fig. 2, lower left corner). Model results apparently suggest that the present-day climate is more favorable for peatland expansion than the mid-Holocene climate since the *f*_P_ simulated for mid-Holocene climate nowhere exceeds the present level except in the Tobol river basin (i.e., between 56*–*58N and 65*–*70E).

The climate for 2100 under the RCP 8.5 scenario suggests unprecedented changes in the geographic pattern of *f*_P_ (Fig. 2, upper right corner): a dramatic decrease to the south of 60N and an impressive increase to north of 60N. According to this scenario, the 60^th^ parallel will divide Western Siberia into sharply distinct climate zones. In the wet climate zone, to the north of 60N, most land becomes waterlogged, whereas in the dry climate zone, to the south of 60N, most peatlands will experience difficulties in maintaining their moisture balance.

## Discussion

Using simple climatic indices such as WPE to delineate the potential geographic distribution of boreal peatlands does not look like a very novel approach. Nevertheless, we seemingly were the first who theoretically justified the use of WPE for defining a climatic threshold for peatland expansion. Somewhat similar climate characteristics, *P*_w_ – PET, were used by Gignac *et al.*^33^ to define the climatic threshold below which *Sphagnum*-dominated peatlands cannot occur. The negative value of the threshold, −60 mm/yr, that they found empirically, agrees with our assumption that *P*_w_ exceeds evapotranspiration at the critical depth of soil moisture in the regions suitable for peatland expansion. Since evapotranspiration at such a level of the water table is not as high as PET, *P*_w_ – PET might be negative. For example, the threshold *P*_w_ – PET = −60 mentioned above is equivalent to the threshold WPE = 60, when PET = 400 mm/yr.

Moreover, the empirically determined value of the *P*_w_ – PET threshold could be interpreted using IDM as the climatic threshold for peatland expansion over watersheds where P/PET = 1.2 and 10 < *g* < 15 m (Fig. 2, lower right corner). The major advantage of IDM-based thresholds over the empirical thresholds is that IDM takes into account the effects of landscape morphology. The low elevation of watersheds above the level of rivers and lakes is an important factor of peatland expansion over Western Siberia. IDM allows us to quantify the effect of this factor on the climatic thresholds.

The results of our study contribute to the understanding of the climatic thresholds for switching between peatland expansion and peatland degradation, and call for further research. More accurate quantification of the effects of landscape morphology on peatland expansion requires data on the spatial distribution of the drainage network density and the average watershed elevation above the level of the drainage streams. Such data could probably be derived from the next generation of digital elevation models (DEMs) that would provide sufficient accuracy of elevation estimates^34^.

It is worth noting at this point that IDM simulates the fraction of the land area suitable for peatland expansion (or initiation), not the fraction of peatlands. Lateral expansion of peatlands is a process that takes hundreds of years. It should be simulated with models that take into account the rate of peat accumulation. The IDM could be used to evaluate the potential either for peatland initiation or for their further expansion under a given climate.

The IDM could be also used to evaluate the risk of peatlands degradation. A drying trend suggested by the RCP 8.5 scenario for 2100 in the regions located to the south of 60N challenges the hypothesis of the so-called “aggressive” paludification of Western Siberia. This hypothesis, based on the observed increase in peatland area, leads to the conclusion that peatlands may cover all of the middle and southern taiga belt during the next 1000 years (ref. 18). The “aggressive” paludification can, however, be arrested or reversed by climate change. The drying trend mentioned above implies a water table reduction in peatlands, which are widespread within the southern taiga belt, and their gradual degradation. A reduced water table not only turns a peatland into a carbon sink but also makes it more vulnerable to burning. Moreover, according to experimental research^35^, a reduced water table dramatically amplifies carbon losses during wildfire – that is, peatland wildfires may have unprecedented consequences in the future.

The climate changes that took place in the past might have had a similar effect on paludification in some regions. Comparing mid-Holocene and present-day climate we found a dramatic decline in *f*_P_ within the Tobol river basin. This calls for a field study: evidence that peatlands in this area really did degrade over that time would be the best substantiation for the hypothesis about the possible degradation of peatlands that may occur in the southern taiga belt of Western Siberia under an RCP 8.5 scenario for 2100 climate.

## Methods

The impeded drainage model (IDM) which is used here is based on the Dupuit-Forchheimer theory of groundwater movement (aka hydraulic theory) and a few additional assumptions (see Supplementary Information) that allow us to reduce the number of parameters quantifying landscape morphology and to derive the equation for calculating *f*_P_ , the fraction of land suitable for peatland expansion:


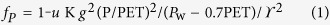


where P is the annual amount of precipitation, in mm/yr; PET is the potential evapotranspiration, in mm/yr; *P*_w_ is precipitation during the warm period of the year, in mm/yr; K is hydraulic conductivity, in m/yr; *g* is the averaged watershed elevation above the level of the drainage system, in m; *ϒ* is the coefficient, in km, that parameterizes the average distance between drainage streams based on the empirical equation proposed by Wang and Wu^36^; *u* is a conversion factor (that is, the factor applied for matching variable dimensions).

PET is calculated from the monthly temperature simulated by MPI-ESM (refs 20, 21) using the Thornthwaite method^37^. The monthly precipitation simulated by the same model is used to calculate P and *P*_w_. K is calculated as K_0_ N_w_/12, where N_w_ is the length of the warm period, in months, and K_0_ = 350 m/yr (this corresponds to about 10^−5^ m/s and falls within the empirically established range of typical values for peat and mineral ground^38^). *ϒ i*s equal to 1.1 km, *u* = 0.001, and *g* is estimated by using model optimization (see Supplementary Information) to fit the contemporary distribution of peatlands.

Model optimization leads to the following equation for making projections of *f*_P_ based on its present-day value and relative changes in P, PET, and WPE:


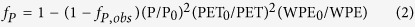


where *f*_*P,obs*_, P_0_, PET_0_, and WPE_0_ are the present day values of *f*_*P*_, P, PET, and WPE, respectively. The domain of application of this equation is restricted to the cells of geographic grids where 0 < *f*_*p,obs*_ < 1, WPE_0_ > 0, WPE > 0, PET_0_ > 0, and PET > 0.

The fields of mean monthly temperature and precipitation used in this study were simulated by the Max Planck Institute - Earth System Model (MPI-ESM) for use in the Coupled Model Intercomparison Project Phase 5 (CMIP5) and in the Paleoclimate Model Intercomparison Project Phase 3 (PMIP3) (refs 20, 21). Since Earth System Models are not perfect, the model-based reconstructions of mean monthly temperature and precipitation are suggestive and not conclusive. Consequently, the results of our study are of the same nature. They are to provide a heuristic point of view for interpretation of the existing data and for setting research questions for the further field studies.

## Additional Information

**How to cite this article**: Alexandrov, G. A. *et al.* The influence of climate on peatland extent in Western Siberia since the Last Glacial Maximum. *Sci. Rep.* **6**, 24784; doi: 10.1038/srep24784 (2016).

## Supplementary Material

Supplementary Information

## Figures and Tables

**Figure 1:**
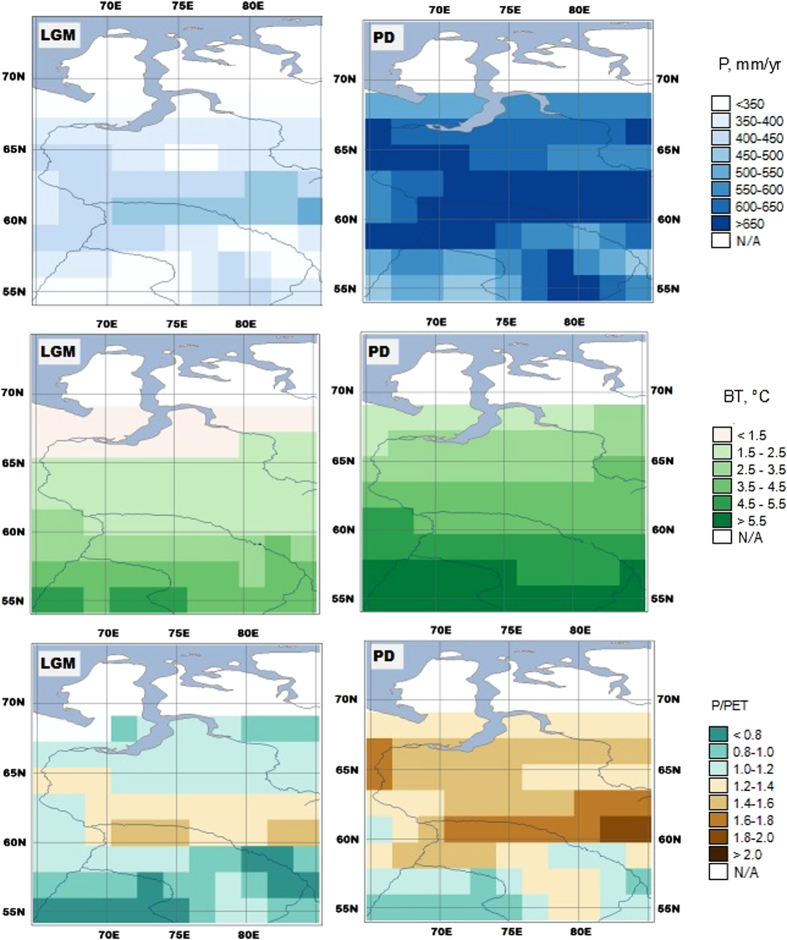
Western Siberia climate during LGM (left) and at present (right) derived from the monthly precipitation and monthly temperature simulated by the MPI-ESM (refs 20, 21). P is annual precipitation, in mm/yr. BT is biotemperature (ref. 22), the sum of positive monthly temperatures divided by 12. P/PET is the ratio of annual precipitation to potential evapotranspiration calculated from monthly temperature using the Thornthwaite method (ref. 37). Made with *MapWindow* 4.8.8 (http://www.mapwindow.org/), *Natural Earth* public domain map data (http://www.naturalearthdata.com/about/terms-of-use/), and map colors from www.ColorBrewer.org.
