# Supplementary material for: The influence of climate on peatland extent in Western Siberia since the Last Glacial Maximum
Source: Sci Rep. 2016 Apr 20;6:24784. doi: 10.1038/srep24784 (PMC4837369; doi:10.1038/srep24784)
Supplement: Supplementary Information [file srep24784-s1.doc]

The influence of climate on peatland extent in Western Siberia since the Last Glacial Maximum

G.A. Alexandrov^1^, V.A. Brovkin^2^, T. Kleinen^2^

^1^ A.M. Obukhov Institute of Atmospheric Physics, Russian Academy of Sciences, Pyzhevsky 3, Moscow, 119017, Russia

^2^ Max Planck Institute for Meteorology, Bundesstrasse 53, 20146 Hamburg, Germany

Correspondence should be addressed to G.A.A. (g.alexandrov@ifaran.ru)

**Supplementary Methods**

## The basics of impeded drainage model

The steady-state water table, *h,* in ground with hydraulic conductivity equal to K, satisfies the equation:

(S1)

where U is effective rainfall, the part of precipitation that percolates into drainage streams as groundwater runoff, and *a* is the half-distance between the drainage streams.

This equation has the following solution:

(S2)

This could easily be checked by substituting equation (S2) to equation (S1).

The water table reaches the maximum height at the drainage divide, that is, at x=0:

(S3)

In the case of a flat ground surface, the minimal depth of the water table occurs at the drainage divide. It is equal to *g*-*h*_max_.

Strictly speaking, the impeded drainage model assumes that *g* > *h*_max_. To lift this model restriction one can assume that K does not remain constant but increases rapidly when the water table is approaching the level of the ground surface. Then the movement of water in the parts of the watershed where the water table is above the ground level can be considered a “quasi-subsurface runoff”.

Another way is to assume that the water table is kept below ground level by very quick surface runoff:

(S4)

where *x*_0_ is the point of intersection of *h* and *g*:

(S5)

given by equation

(S6)

Then, assuming some symmetry in the sense of the distance to draining streams, one may estimate the fraction of the watershed area where the water table is close to the ground surface as the ratio of *x_0_^2^* to *a^2^*:

(S7)

The area where the water table is close to the ground surface is the area which is suitable for peatland initiation. Therefore, we consider *f*_P_ as an estimate of the fraction of land suitable for peatland initiation under given climatic conditions (U) and landscape morphology (*g* and *a*).

## Climatic drivers of draining streams density

The empirical relationship between the perennial stream density, D_P_, and PET/P (Wang & Wu, Hydrol. Earth Syst. Sci., 17, 315–324, 2013) is:

(S8)

This allows us to make an assumption about the geographic distribution of one of the model parameters related to landscape morphology. Assuming that *a*=1/(2D_P_), we come to an equation that links *a* and PET/P:

(S9)

where *ϒ*=1.1 km. This turns equation (S7) to

(S10)

The assumed relationship between *a* and D_P_ is based on the idea that D_P_ is equal to the ratio of stream length to the area of land around the stream that spreads from the left drainage divide to the right drainage divide, that is, between the boundaries of the drainage basin. In this case the area around an approximately straight piece of the stream is equal to the length of this piece, L, multiplied by the distance between left and right drainage divide, which is equal to 2*a*, and so D_P_=L/(L 2*a*)=1/(2*a*), and thus *a*=1/(2D_P_).

## Critical norm of actual evapotranspiration

The amount of effective rainfall, U, depends not only on the precipitation during the warm period, P_w_, but also on the amount of actual evapotranspiration (AET). We assume that actual evapotranspiration corresponds to the critical norm – 0.7PET, which is observed in *Sphagnum*-dominated peatlands when the water table is at a depth of 30-40 cm. This depth of water table is critical: a further lowering of the water table is incompatible with stable *Sphagnum* growth. Assuming that U= P_w_– 0.7PET in the equation (S10), we obtain the estimate of the watershed fraction that could be covered by 30-cm thick layer of peat.

## Model optimization

Model optimization is the technique which is used to determine the value of a model parameter when its value cannot be measured with sufficient accuracy. In such a case the observed values of the modelled entity are considered as indirect measurements of this parameter. Here we use data on the present distribution of peatland area as an indirect measurement of the average watershed elevation above the level of the drainage system (*g*)*.*

According to the engineering geological map of V. T. Trofimov (Kainozoyskie otlojeniya, pochvy, merzlotnye i injenerno-geologicheskie usloviya Zapadnoi Sibiri, Moscow State University, 1980, pp. 128-151) reproduced in the book “Bolotnye systemy Zapadnoi Sibiri” by Liss et al. (Moscow State University, 2001), *g* is seldom more than 25 m and often is less than 10 m in major parts of Western Siberia. In these flat featureless landscapes, where a 5-meter difference in the estimates of *g* may lead to 50% difference in the estimate of *f*_P_ (the fraction of land suitable for peatlands), *f*_P_ estimates are sensitive to relative height error. This makes the use of digital elevation models (DEMs) a challenging task: the most accurate DEM that will be available in the near future would provide relative heights with 2-meter accuracy for 12x12 m raster (Rizzoli et al., Relative height error analysis of TanDEM-X elevation data. ISPRS J. Photogramm. Remote Sens. 73, 30–38 , 2012). Meanwhile, it seems more reasonable to derive *g* from the present distribution of peatlands, as reported by Peregon et al. (*J. Geophys. Res. Biogeosciences* **113,** G01007, 2008) on a half-degree grid.

The model optimization procedure consists of two steps. In the first step, we estimate the fraction of peatland area, *f*_P,obs_, within MPI-ESM grid cells. For each MPI-ESM grid cell we calculate the total wetland area and the total land area in the half-degree cells that fall within the boundaries of the MPI-ESM grid cell and then divide the total wetland area by the total land area.

As second step, we calculate the optimal *g* values, *g*_opt_ , for each MPI-ESM grid cell, using the equation

(S11)

where *f*_P,5_ is *f*_P_ estimate calculated using IDM for *g*=5m, and assuming that *f*_P,5_ >0.

In most cases *f*_P,5_ =0, when WPE is less or equal to zero. However, where *g<* 5m, *f*_P,5_ could be equal to zero under positive WPE. In such cases, we determine *g*_opt_ using a slightly modified equation:

(S12)

where

(S13)

The *g*_opt_ thus calculated varies between 2.6 and 19.6 meters (Fig S1), that is, within the range implied by the Trofimov map mentioned above.

As can be seen in Fig S1, *f*_P_ calculated for *g*=*g*_opt_ and present climate is equal to *f*_P,obs_ everywhere, except in the cells, where WPE is not positive. Calculating *f*_P_ for *g*=*g*_opt_ and LGM, mid-Holocene, or future climate we take into account not just the changing climatic conditions, but also the constraints imposed by landscape morphology.

For example, there are two regions in the map of WPE calculated for LGM climate, Fig S2, that could be suitable for peatlands expansion if there were no constraints imposed by landscape morphology. Both are in the 60-62N latitudinal belt. One is between 72 and 76E, and another is between 82 and 86E.

The model optimization makes the *f*_P_ estimates quite robust to possible bias in climate projections, because the estimates depend on the relative changes in P, WPE and PET, not on their absolute values: substituting *g*_opt_ into Equation (S10) gives

$f_{P}=1-(1-f_{p, obs})\left(\frac{P}{P_{0}}\right)^{2}\left(\frac{PET_{0}}{PET}\right)^{2}\left(\frac{WPE_{0}}{WPE}\right)$ (S14)

where P_0_, PET_0_, and WPE_0_ are the present day values of P, PET, and WPE, respectively, 0<f_p,obs_<1, and 0<f_p,5_<1.

WPE alone allows us to delineate the regions where climatic factors may prevent a massive expansion of peatlands despite favourable landscape morphology. However, comparing the WPE map for present-day climate, Fig S2, and the map of the contemporary peatland distribution, Fig S1, one can see that a negative WPE does not prevent peatland expansion in the regions adjacent to the Gulf of Ob. Within the framework of the impeded drainage model, this fact could be explained as follows.

The negative WPE prevents peatland expansion over the parts of a watershed that rise above the level of the draining streams. The parts of the watershed that lie at or below the level of the draining streams could be occupied by peatlands. Thus kettle ponds which are widespread in the regions adjacent to the Gulf of Ob may evolve to peatlands in a process of terrestrialization.

There are two processes, paludification and terrestrialization, that lead to peatland expansion in a given region. The impeded drainage model deals with the paludification process that may lead to massive expansion of peatlands over the slopes and hillocks under some climatic conditions. Strictly speaking, it is not applicable to the watersheds where the average elevation above the draining streams is too low, that is, where *g*<2.5m. In such watersheds, even relatively small depressions may reach the level of the draining streams, and so the fraction of land that could be covered by peatlands depends primarily on the area of the watershed covered by depressions of sufficient size.

Applying the impeded drainage model to a particular watershed one should take into account the areas occupied by hillocks and hollows. The average elevation above the draining streams gives us the approximate shape of the water table, Equation (S2), but to calculate the distance from surface of the water table to the ground surface we have to know the shape of the ground surface. Neglecting the effects of the spatial variations in the elevation above the draining streams may lead to an underestimation of the watershed fraction suitable for peatland expansion under low WPE (some hollows could be suitable for peatland expansion). And under high WPE, this may lead to overestimation of this fraction (some hillocks could be unsuitable for peatland expansion).

Applying the impeded drainage model at the scale of an MPI-ESM grid cell, we may assume that the effects of spatial variations are not as strong as at the scale of a particular watershed. Errors resulting from neglecting the pattern of hillocks and hollows at various watersheds could be of opposite sign and may partly cancel each other at this scale. Nevertheless, we cannot assume that *f_p_* projections have a high accuracy, especially in the case of dramatic changes in WPE. To evaluate the accuracy of the proposed method we need “a second point”, that is, some data on the climate and on the distribution of peatlands during some period in the past. Meanwhile, *f_p_* projections should be taken as suggestive, not as conclusive. They serve to propose an independent interpretation of the data obtained by the analysis of peat cores and a hypothesis that could be checked by further field studies in the Tobol river basin.


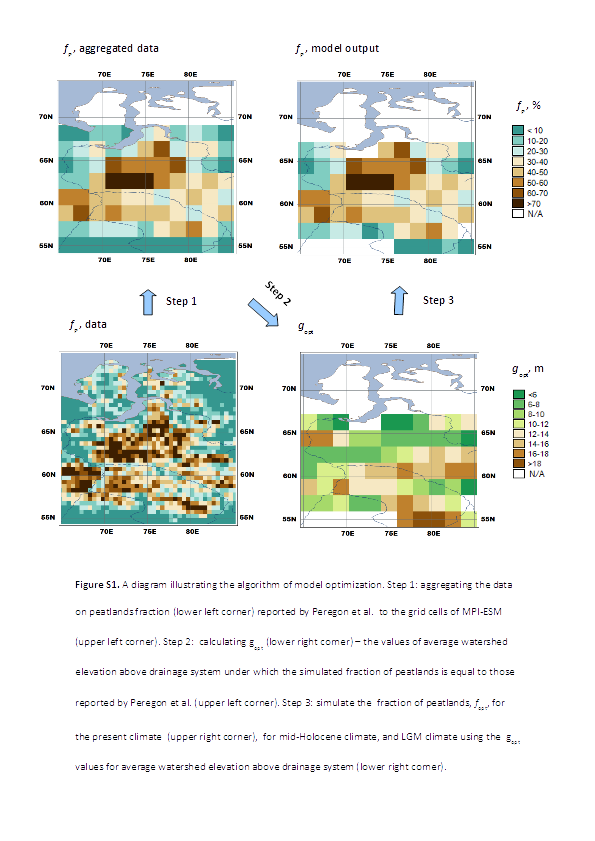


**Figure S1.** A diagram illustrating the algorithm of model optimization. Step 1: aggregating the data on peatlands fraction (lower left corner) reported by Peregon et al. to the grid cells of MPI-ESM (upper left corner). Step 2: calculating g_opt_ (lower right corner) – the values of average watershed elevation above drainage system under which the simulated fraction of peatlands is equal to those reported by Peregon et al. (upper left corner). Step 3: simulate the fraction of peatlands, *f*_opt_, for the present climate (upper right corner), for mid-Holocene climate, and LGM climate using the g_opt_ values for average watershed elevation above drainage system (lower right corner).

Made with *MapWindow* 4.8.8 (<http://www.mapwindow.org/>), *Natural Earth* public domain map data (<http://www.naturalearthdata.com/about/terms-of-use/>), and map colors from [www.ColorBrewer.org](http://www.ColorBrewer.org/).


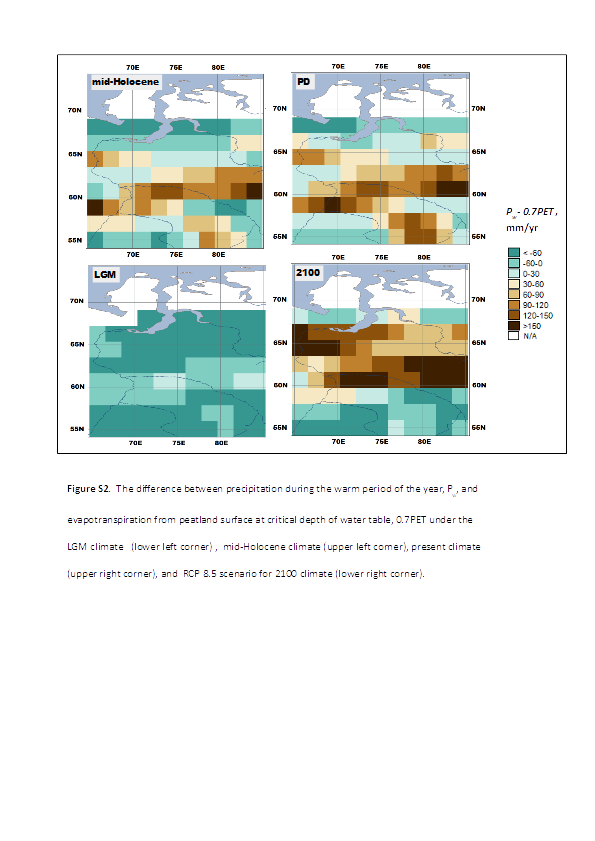


**Figure S2**. The difference between precipitation during the warm period of the year, P_w_, and evapotranspiration from peatland surface at critical depth of water table, 0.7PET, under the LGM climate (lower left corner) , mid-Holocene climate (upper left corner), present climate (upper right corner), and RCP 8.5 scenario for 2100 climate (lower right corner).

Made with *MapWindow* 4.8.8 (<http://www.mapwindow.org/>), *Natural Earth* public domain map data (<http://www.naturalearthdata.com/about/terms-of-use/>), and map colors from [www.ColorBrewer.org](http://www.ColorBrewer.org/).
